# Is age an effect modifier in the relationship between HAM / TSP and depression?

**DOI:** 10.1186/1742-4690-11-S1-P25

**Published:** 2014-01-07

**Authors:** Ney Boa-Sorte, Ana Verena Galvão-Castro, Danilo Borba, Maria Fernanda Rios Grassi, Bernardo Galvão-Castro

**Affiliations:** 1 Centro HTLV/ Escola Bahiana de Medicina e Saúde Publica, Brazil; 2 Universidade Federal da Bahia, Brazil; 3 LASP, Fiocruz/BA, Brazil

## 

It is estimated that depression occurs in approximately 30% of people living with HTLV-1. Nevertheless, the association between depression and TSP/HAM is still controversial. We have investigated if age plays a role in the association between HAM/TSP and depression among people living with HTLV-1 from Salvador, Brazil. A convenience sample was selected and 107 people infected with HTLV-1 (ELISA and W.Blot): asymptomatic = 60, (56.1%) and HAM / TSP = 47, (43.9%), were enrolled in a cross-sectional study conducted between March and November 2009 at the HTLV reference Center Bahiana School of Medicine. The instruments employed were a questionnaire, used for the collection of Clinical and epidemiological data and the Mini International Neuropsychiatric Interview, Brazilian Version 5.0.0 (M.I.N.I.), for the diagnosis of depression. HAM/TSP was classified according to ascertainment level (probable and definite) criteria. Prevalence ratios (PR) were calculated according to gender, age, marital status, ethnicity, income and education. To analyze the effect modification, we used the homogeneity test Mantel-Haenzael. The prevalence of depression was 39.4 % (41/107). Overall, no significant association was observed between HAM / TSP disease and major depression. However, we found a positive association between HAM / TSP and major depression among patients aged 18 to 39 years compared to individuals >40 years (PR: 2.59, 95% CI :1.36-4. 95). The association between HAM/TSP and depression, in this age group, may occur because younger patients may be more affected by HAM / TSP symptom such as gait difficulties, erectile dysfunction and sphincter disturbances.

